# Perpendicular Magnetic Anisotropy Engineering for Skyrmion Thermal Stability and Pulsed-Current-Driven Dynamics in a Z-Junction Nanotrack

**DOI:** 10.3390/nano16211432

**Published:** 2026-11-05

**Authors:** Mohammed Al Bahri, Salim Al-Kamiyani, David Laroze, Alexis Matheu, Tamara González-Vega, Eduardo Saavedra, Felipe Tejo

**Affiliations:** 1 Department of Basic and Applied Sciences, A’Sharqiyah University, P.O. Box 42, Ibra PC 400, Oman; 2 Instituto de Alta Investigación, Universidad de Tarapacá, Arica 1000000, Chile; 3 Centro de Investigación Institucional, Universidad Bernardo O’Higgins, Santiago 8370993, Chile; 4 Facultad de Ciencias de la Vida, Universidad Viña del Mar, Agua Santa 7055, Rodelillo, Viña del Mar 2572007, Chile; 5 Department of Physics, University of Santiago de Chile, Av. Victor Jara 3493, Santiago 9170124, Metropolitan Region, Chile; 6 Vicerrectoría de Investigación, Innovación y Postgrado, Universidad Central de Chile, Toesca 1783, Santiago 8370178, Metropolitan Region, Chile

**Keywords:** magnetic skyrmion, perpendicular magnetic anisotropy, thermal stability, Z-junction nanotrack, micromagnetic simulation, topological charge, skyrmion annihilation, spintronic devices

## Abstract

Magnetic skyrmions are promising information carriers for nanoscale spintronic devices, but their use in confined structures requires control of both topological survival and current-driven motion. We investigate perpendicular magnetic anisotropy engineering in a Z-junction nanotrack using micromagnetic simulations. The anisotropy is varied from 0.70 to 1.00 MJ/m^3^ at 300 K for a junction depth of 50 nm, junction width of 5 nm, and pulsed current density of 1.8 × 10^12^ A/m^2^. Five independent thermal realisations are considered for each finite-temperature condition. No annihilation is observed for *K_u_* = 0.70–0.90 MJ/m^3^ during the 63 ns observation interval, whereas all five realisations at 0.95 and 1.00 MJ/m^3^ undergo annihilation within approximately 9–10 ns. The 0.90 MJ/m^3^ case remains topologically intact despite strong compression. Additional in-plane mesh refinements reproduce survival at 0.90 MJ/m^3^ and annihilation at 1.00 MJ/m^3^. The selected compact reference condition *K_u_* = 0.85 MJ/m^3^ is then examined from 0 to 500 K, with a deterministic reference at 0 K. The trajectories show preservation of the skyrmion-like topological state within the available observations, together with temperature-dependent positional, radial, and velocity variations. These results distinguish finite-time topological survival from dynamical reproducibility. The temperature sweep describes thermal-noise effects within a fixed-parameter model; it does not establish a thermally activated lifetime, a universal optimal anisotropy, or an experimental device operating-temperature range.

## 1. Introduction

Magnetic skyrmions are nanoscale, noncollinear spin textures characterized by a continuous rotation of the local magnetization and a nonzero topological charge. Their particle-like behavior, compact dimensions, and ability to be created, transported, and annihilated have attracted considerable interest for future spintronic technologies. Experimental observations of skyrmion lattices in chiral magnets established the physical existence of these topological structures, while direct real-space imaging confirmed their nanoscale magnetic configuration [1,2]. Their topologically nontrivial character can provide enhanced robustness against weak perturbations, making skyrmions promising information carriers for high-density memory, logic, and unconventional computing devices. Selective excitation of spin-wave modes in skyrmion clusters has also been proposed for magnonic logic devices [3].

The feasibility of skyrmion-based devices depends on the reliable nucleation and controlled motion of isolated skyrmions in confined magnetic nanostructures. The stray field of a magnetic tip can also control the nucleation and annihilation of Néel skyrmions confined in nanodisks [4]. Micromagnetic studies have shown that isolated skyrmions can be stabilized and driven along ultrathin magnetic nanotracks by spin-polarized currents [5]. In these systems, the skyrmion state results from a balance among the exchange interaction, perpendicular magnetic anisotropy, demagnetizing energy, and interfacial Dzyaloshinskii–Moriya interaction. The DMI favors a chiral rotation of the magnetization and modifies the boundary conditions at the nanotrack edges, whereas perpendicular magnetic anisotropy promotes out-of-plane magnetization and strongly influences the equilibrium skyrmion size [6]. The relative strengths of these interactions therefore determine whether the skyrmion remains stable, becomes strongly compressed, or collapses into the uniformly magnetized state. For cobalt nanodots without DMI, analytical and numerical phase diagrams have shown that anisotropy and an applied field control the stability region of Bloch skyrmions [7].

Important experimental advances have demonstrated both the generation and current-driven manipulation of magnetic skyrmions. Jiang et al. generated skyrmion bubbles at room temperature by applying an inhomogeneous current through a geometrical constriction [8]. Stable skyrmions and their current-driven motion were subsequently observed in ultrathin metallic ferromagnets with perpendicular magnetic anisotropy [9]. Room-temperature Néel-type skyrmions were also demonstrated in sputtered Pt/Co/MgO nanostructures at zero external magnetic field, highlighting the important role of interfacial DMI and magnetic-energy optimization in stabilizing the skyrmion state [10]. These findings established ultrathin multilayer systems as promising material platforms for room-temperature skyrmion devices.

Despite these advances, reliable skyrmion transport in a confined nanotrack remains challenging. Depending on the driving torque and confinement, current-driven skyrmions can develop both longitudinal and transverse motion. The resulting skyrmion Hall effect deflects the skyrmion toward one side of the nanotrack and increases the probability of edge interaction and annihilation [11]. This problem becomes more pronounced in narrow tracks, curved sections, junctions, and other geometrically nonuniform regions, where the local confinement potential can substantially modify the trajectory and deformation of the skyrmion. Time-resolved magnetic imaging has directly demonstrated the transverse deflection of current-driven skyrmions and shown that their dynamic response cannot always be described using a perfectly rigid-particle approximation [11]. Internal dynamics have also been studied through the dynamic susceptibility of skyrmion clusters in Co/Pt nanodots [12]. In Co/Pt multilayer nanodots, perpendicular spin-polarized current pulses can excite damped breathing oscillations during the relaxation of skyrmion clusters [13].

Geometrical engineering has consequently emerged as an important approach for controlling skyrmion motion. Local potential barriers can guide the skyrmion and reduce unwanted edge annihilation, although strong confinement can also compress and deform the magnetic texture [14]. Similarly, stepped and constricted nanowires can produce pinning, depinning, transmission, or annihilation depending on the geometrical dimensions and magnetic material parameters [15]. These results show that a geometrical discontinuity does not act merely as a passive obstacle; instead, it creates a nonuniform magnetic-energy landscape that interacts with the skyrmion size, trajectory, and internal structure. Therefore, the design of junction-based nanotracks requires simultaneous optimization of both the device geometry and the intrinsic magnetic properties.

Temperature introduces a further limitation because thermal fluctuations continuously perturb the local magnetization, skyrmion boundary, and current-driven trajectory. Although the nonzero topological charge provides a degree of robustness, this protection is not absolute in finite magnetic nanostructures. Calculations of minimum-energy transition paths have demonstrated that skyrmions can be destroyed through several mechanisms, including internal collapse, singularity-mediated destruction, and boundary-assisted annihilation [16]. Among these mechanisms, escape through a sample boundary can involve a comparatively low energy barrier [17], making edge interaction particularly important for room-temperature nanotrack operation. Thermal agitation can therefore promote irreversible collapse when a current-driven skyrmion is compressed or displaced toward a junction boundary.

The thermal stability of a skyrmion is also closely connected to its magnetic properties and dimensions. Previous micromagnetic simulations showed that changes in perpendicular magnetic anisotropy, saturation magnetization, DMI strength, and nanowire geometry can substantially modify skyrmion lifetime and stability under finite-temperature conditions [18]. In particular, perpendicular magnetic anisotropy is a critical design parameter because it controls the energetic preference for out-of-plane magnetization and modifies the size of the reversed skyrmion core. A moderate increase in *K_u_* may produce a more compact skyrmion, which is desirable for high-density devices; however, excessive anisotropy can reduce the skyrmion size and its ability to accommodate current-induced and thermally induced deformation. Thus, increasing *K_u_* does not necessarily produce a monotonic enhancement of stability, especially when the skyrmion interacts with a confined geometrical region.

Previous work has established that magnetic anisotropy, saturation magnetisation, DMI, temperature, and nanowire dimensions influence skyrmion nucleation and thermal stability [18]. Separately, chirality-dependent pinning and depinning of transverse domain walls have been studied in Z-junction nanowires [19]. These studies do not determine how a skyrmion responds to the combined effects of anisotropy-controlled compression, pulsed-current excitation, and asymmetric confinement in a Z-junction.

The present work examines this coupling using the topological charge, survival and annihilation times, core size, trajectories, and magnetic configurations. In particular, it distinguishes a strongly compressed skyrmion that survives during the observation interval from a configuration that loses its topology. The temperature-dependent analysis further examines how topological preservation can coexist with changes in confined motion and internal deformation. These coupled structural, topological, and dynamical responses are the focus of the investigation.

## 2. Theoretical Model and Simulation Methodology

### 2.1. Micromagnetic Model

The magnetization dynamics were investigated using the GPU-accelerated MuMax3 micromagnetic simulation package, which employs a finite-difference discretization to solve the time- and space-dependent magnetization dynamics [20]. MuMax3 has been extensively verified against analytical solutions and standard micromagnetic problems and is widely used to investigate current-driven skyrmion dynamics in confined magnetic nanostructures.

The local magnetization was represented by the normalized vector

(1) $$m\left(r,t\right)=\frac{M\left(r,t\right)}{M_{s}},$$

where **M** is the magnetization and Ms is the saturation magnetization. Its temporal evolution was described using the Landau–Lifshitz–Gilbert equation, including the adiabatic and nonadiabatic spin-transfer-torque terms [21,22,23,24]:

(2) $$\frac{\partial m}{\partial t}=-\gamma_{0}m\times H_{\mathrm{eff}}+\alpha m\times \frac{\partial m}{\partial t}-\left(u\cdot \nabla \right)m+\xi m\times \left[\left(u\cdot \nabla \right)m\right].$$

Here, *γ*_0_ is the gyromagnetic coefficient in the *H*-field convention, with units m/(A s), *α* is the Gilbert damping constant, and *ξ* is the nonadiabaticity parameter. The spin-drift velocity is given by

(3) $$u=\frac{g\mu_{B}P}{2eM_{s}}J,$$

where *g* is the Landé factor, *μ_B_* is the Bohr magneton, *P* is the spin polarization, *e* is the elementary charge, and **J** is the applied current-density vector.

The effective magnetic field was obtained from the functional derivative of the total magnetic energy:

(4) $$H_{\mathrm{eff}}=-\frac{1}{\mu_{0}M_{s}}\frac{\delta E}{\delta m}+H_{\mathrm{th}},$$

where *μ*_0_ is the vacuum permeability and **H**th is the stochastic thermal field. The total magnetic energy was expressed as

*E* = *E*_ex_ + *E*_ani_ + *E*_DMI_ + *E*_demag_ + *E_Z_*. (5)

The corresponding energy density can be written as

(6) $$\begin{array}{c} \varepsilon =A\sum_{i=x,y,z}{\left(\nabla m_{i}\right)}^{2}+K_{u}\left(1-m_{z}^{2}\right)+D[m_{z}\left(\nabla \cdot m\right)-\left(m\cdot \nabla \right)m_{z}]\\ -\frac{\mu_{0}M_{s}}{2}H_{d}\cdot m-M_{s}B_{\mathrm{ext}}\cdot m \end{array}$$

Here, *A* is the exchange stiffness, *K_u_* is the perpendicular magnetic anisotropy constant, *D* is the interfacial DMI constant, **H***_d_* is the demagnetizing field, and **B**_ext_ is the externally applied magnetic flux density. The interfacial DMI favors the formation of Néel-type skyrmions and strongly affects their size, chirality, confinement, and boundary interaction [6].

Previous theoretical studies have demonstrated that the behavior of current-driven skyrmions differs considerably in confined and constricted geometries because of their interaction with the device boundaries and nonuniform potential landscape [25,26]. Similar micromagnetic approaches have been used in our previous investigations of skyrmion thermal stability, geometric pinning, magnetic-property control, and current-driven dynamics in stepped, constricted, and Z-junction nanowires [18,19,27,28,29,30,31].

### 2.2. Nanotrack Geometry and Numerical Discretization

**Figure 1:**
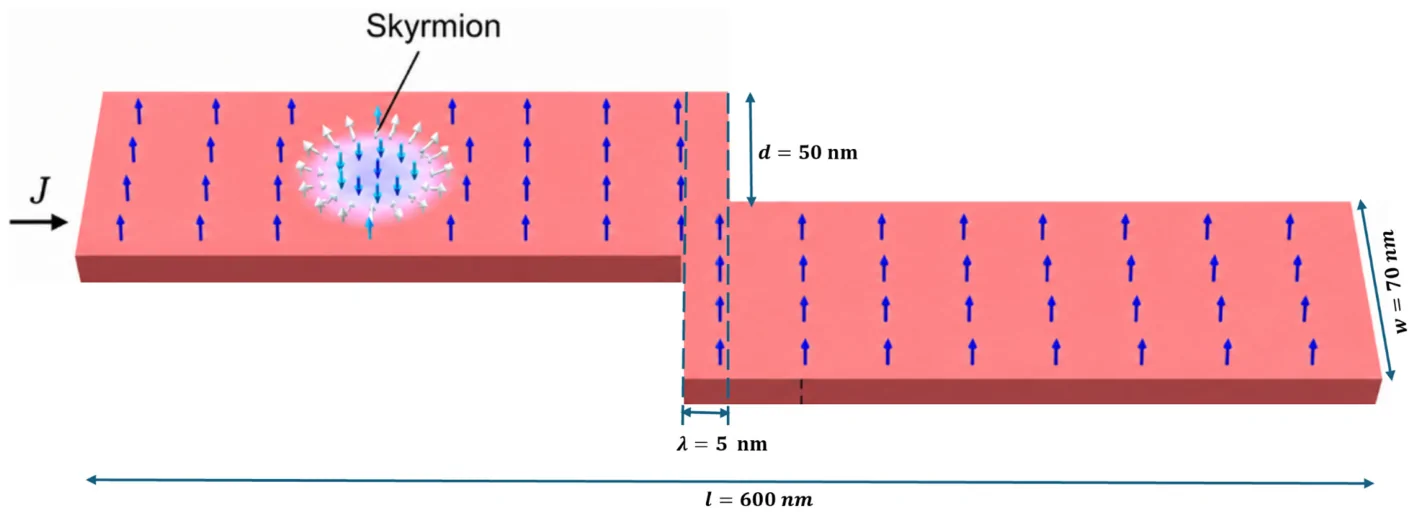
Three-dimensional schematic of the Z-junction nanotrack with total length *l* = 600 nm, arm width *w* = 70 nm, junction depth *d* = 50 nm, and nominal junction width *λ* = 5 nm. The computational box is 600 × 140 × 4 nm^3^. The skyrmion is initially located in the upper arm. The arrow *J* indicates the prescribed driving direction, and the blue symbols represent the perpendicular background magnetisation.

The simulated structure consisted of a perpendicular-magnetic-anisotropy nanotrack containing a Z-shaped junction, as shown in Figure 1. The total computational dimensions were 600 × 140 × 4 nm^3^. The junction depth and junction width were fixed at *d* = 50 nm and *λ* = 5 nm, respectively. The geometric discontinuity between the upper and lower sections of the nanotrack generated an asymmetric confinement region that controlled the current-driven skyrmion motion.

The computational region was divided into 300 × 70 × 2 finite-difference cells, with an individual cell size of 2 × 2 × 2 nm^3^. The exchange length was estimated from

(7) lex=2Aμ0Ms2≈4.6 nm.

To assess sensitivity to the in-plane discretisation, additional simulations were performed with cell sizes of 1.0 × 1.0 × 2.0 nm^3^ and 0.5 × 0.5 × 2.0 nm^3^ and compared with the original 2.0 × 2.0 × 2.0 nm^3^ mesh. Supplementary Figure S1 shows close agreement in the topological-charge evolution for *K_u_* = 0.90 and 1.00 MJ/m^3^. All three meshes preserve the skyrmion-like plateau at 0.90 MJ/m^3^ and reproduce irreversible annihilation at approximately 9–10 ns at 1.00 MJ/m^3^. Thus, the survival and annihilation outcomes in these tested cases are robust against a fourfold reduction in the in-plane cell width. This comparison concerns *Q*(*t*) and the observed topological outcome; it does not independently establish convergence of the minimum equivalent radius.

The geometry was imported into MuMax3 using an image-defined shape. Open (non-periodic) boundary conditions were applied along all spatial directions, without periodic replication of the computational domain. This approach preserved the physical Z-junction boundaries and was consistent with the geometrical construction procedures used in our previous studies of stepped and Z-junction magnetic nanowires [19,27,31].

The material parameters were fixed at

*M_s_* = 8.75 × 10^5^ A/m, *A* = 1.0 × 10^−11^ J/m, (8)

*D* = 1.5 × 10^−3^ J/m^2^, *α* = 0.05. (9)

The perpendicular easy axis was oriented along **u**_ani_ = (0,0,1). A uniform stabilizing magnetic flux density **B**_ext_ = (0,0,0.1) T was applied along the +*z* direction. The perpendicular magnetic anisotropy was systematically varied as

*K_u_* = 0.70, 0.75, 0.80, 0.85, 0.90, 0.95, 1.00 MJ/m^3^, (10)

while the remaining material, geometrical, thermal, and current parameters were held constant. Systematic variation intrinsic magnetic properties has previously been shown to provide an effective means of controlling the thermal stability, size, mobility, and pinning of topological magnetic structures [28,29,30,31,32].

The adaptive third-order Runge–Kutta solver was used, with the numerical settings MaxErr = 5 × 10^−6^ and MaxDt = 1 × 10^−12^ s.

The material is treated as an idealised ferromagnet with perpendicular anisotropy and interfacial DMI. For comparison, the experimental Ir/Co/Pt study of Moreau-Luchaire et al. used *M_s_* = 956 kA/m and *K_u_* = 0.717 MJ/m^3^ in its micromagnetic analysis, assumed A = 10 pJ/m, and inferred D ≈ 1.9 ± 0.2 mJ/m^2^ from skyrmion-size data [33]. These values support the magnetic scales adopted here, but do not constitute a joint calibration of the complete parameter set, including the 4 nm thickness, damping, and spin-transfer coefficients.

### 2.3. Initial Skyrmion Preparation

A previously stabilized Néel-type skyrmion configuration was loaded into the upper section of the Z-junction nanotrack. To ensure that the simulated response was not affected by artificial initialization transients, the magnetic state was initially relaxed at *T* = 0 K, zero current, and *K_u_* = 0.60 MJ/m^3^.

The anisotropy was increased gradually, with energy relaxation between increments. For example, the *K_u_* = 0.70 MJ/m^3^ state was prepared using the sequence 0.60, 0.62, 0.64, 0.66, 0.68, and 0.70 MJ/m^3^. This preparation was intended to reduce transients associated with an abrupt change in anisotropy. The example specifies the 0.70 MJ/m^3^ schedule; no terminal increment for the other target values is inferred from it.

After reaching the required anisotropy, the system was allowed to evolve for 1 ns at T = 0 K and zero current. This preliminary run confirmed that the skyrmion was stable before thermal fluctuations and the driving current were introduced. The relaxed magnetization configuration was then used as the initial condition for the room-temperature simulations. Gradual initialization and zero-temperature relaxation have also been employed in our earlier investigations of thermally stable skyrmions and magnetic textures [18,27,28,29].

### 2.4. Thermal-Field Implementation

Room-temperature effects were included using the stochastic thermal field implemented in MuMax3. The thermal field was introduced according to the fluctuation–dissipation theorem [34,35]. Its statistical properties satisfy

(11) $$\langle H_{\mathrm{th},i}\left(t\right)\rangle =0$$

(12) $$\left\langle H_{\mathrm{th},i}(t)H_{\mathrm{th},j}(t^{\prime })\right\rangle =\frac{2\alpha k_{B}T}{\gamma_{0}\mu_{0}M_{s}V_{\mathrm{cell}}}\delta_{\mathrm{ij}}\delta \left(t-t^{\prime }\right),$$

where *k_B_* is the Boltzmann constant, *T* is temperature, *V*_cell_ is the simulation-cell volume, and *δ_ij_* and *δ*(*t* − *t*′) are the Kronecker and Dirac delta functions, respectively.

The operating temperature was set to *T* = 300 K. After the zero-temperature preparation, the system was allowed to thermally equilibrate for 1 ns at zero current. An additional zero-current interval of 2 ns was then used to confirm that the thermally equilibrated skyrmion remained stable before the driving pulse was applied.

For each investigated anisotropy at T = 300 K and each nonzero temperature in the temperature-dependent study, five independent MuMax3 simulations were performed using different random seeds for the stochastic thermal field. All realisations used the same material parameters, geometry, initial-state preparation and current-pulse protocol. The observables were evaluated separately for each realisation, and the reported ensemble means and standard deviations were calculated from the five independent results. At T = 0 K, the thermal field vanishes and the deterministic simulation was used as the reference. Thermal-field-based micromagnetic protocols have previously been applied in our studies of domain-wall and skyrmion thermal stability in magnetic nanowires [18,19,27,28,29,30,31].

### 2.5. Pulsed-Current Excitation

Following thermal equilibration and zero-current observation, the skyrmion was driven using a unipolar square pulsed current applied along the positive x direction, **J** = (*J*_max_,0,0), with *J*_max_ = 1.8 × 10^12^ A/m^2^. The spin polarization and nonadiabaticity parameters were set to P = 0.40 and ξ = 0.05, respectively. These parameters determine the strengths of the adiabatic and nonadiabatic spin-transfer torques in the Zhang–Li formulation [23,24,36].

The pulse frequency was fixed at *f* = 1.3 GHz, giving

(13) Tp=1f≈0.769 ns,ton=Tp2≈0.384 ns,toff=Tp−ton≈0.384 ns.

A duty cycle of 50% was used. The pulse train was applied for 50 ns. After removal of the pulse train, the simulation continued for an additional 10 ns at zero current to examine backward relaxation, pinning, survival, or annihilation. Excluding the preliminary zero-temperature stability check, the analyzed room-temperature interval was therefore 1 + 2 + 50 + 10 = 63 ns. The applied-current loop was integrated using a time increment of 1 ps. The simulation table was recorded every 5 ps, while complete magnetization configurations were stored every 200 ps.

Current-driven motion in confined geometries has been investigated using related spin-transfer-torque models in earlier skyrmion studies [5,25,26] and in our previous nanowire simulations [19,27,29,30,31]. At the collective-coordinate level, the motion can also be interpreted using the Thiele formalism, which balances the gyrotropic, dissipative, driving, and confinement forces acting on the magnetic texture [37].

The amplitude *J*_max_ = 1.8 × 10^12^ A/m^2^ lies within the scale explored in theoretical spin-transfer studies [5] and demonstrated in experiments: skyrmions have been moved using 5 ns pulses at 1.8 × 10^12^ A/m^2^ [38], guided at 7.7 × 10^11^ A/m^2^ [39], and driven by subnanosecond pulses in synthetic antiferromagnets [40]. Time-resolved measurements have also used 2.5 ns pulses at 1.2 × 10^12^ A/m^2^ [41]. These precedents concern the current-density scale; the spin–orbit torques and current distributions of those multilayers are not equivalent to the in-plane Zhang–Li spin-transfer model used here.

Joule heating is not included: the thermal-bath temperature is prescribed independently of the applied current. The 50% duty cycle does not imply complete cooling between pulses. Heating could modify the material parameters, pinning, and annihilation response. Its quantitative assessment requires material-specific electrical and thermal properties, interfaces, the substrate, and the local current distribution near the junction.

### 2.6. Evaluation of the Skyrmion Properties

The topological state was characterized using the skyrmion number or topological charge:

(14) $$Q=\frac{1}{4\pi }\int m\cdot \left(\frac{\partial m}{\partial x}\times \frac{\partial m}{\partial y}\right)\;\mathrm{dx}\;\mathrm{dy}.$$

This expression measures the winding of the magnetization over the two-dimensional magnetic film [42]. An intact skyrmion was identified by Q ≈ −1, whereas a topologically trivial state following annihilation was characterized by Q → 0. The topological charge was recorded directly using the MuMax3 quantity ext_topologicalcharge.

The skyrmion center position was obtained from the centroid of the largest connected reversed-core region satisfying *m_z_* < 0. Restricting the analysis to the largest connected region prevents isolated thermally reversed cells from being incorrectly included in the skyrmion position and size calculations. The center coordinates were calculated as

(15) $$x_{\mathrm{sky}}=\frac{1}{N_{-}}\sum_{i=1}^{N_{-}}x_{i},\;y_{\mathrm{sky}}=\frac{1}{N_{-}}\sum_{i=1}^{N_{-}}y_{i},$$

where *N*_−_ is the number of cells belonging to the largest connected reversed-core region and *x_i_*,*y_i_* are their coordinates. The MuMax3 quantity ext_bubblepos was also recorded for comparison. The equivalent radius is the radius of a circle with the same core area:

(16) $$A_{\mathrm{sky}}\;=\;N_{-}\Delta x\Delta y,\;R_{\mathrm{eq}}=\sqrt{\frac{A_{\mathrm{sky}}}{\pi }}.$$

Here *N*_−_ denotes the number of pixels in the selected reversed-core area. The equivalent radius is an area-based measure of a potentially deformed skyrmion, rather than an assumption that its instantaneous shape is circular.

The minimum equivalent radius *R*_eq,min_ was defined as the smallest value reached during the observation interval. For the annihilated cases it represents the smallest detectable connected core immediately before complete topological collapse. Similar area and topological-stability analyses were used in our previous thermal-skyrmion study [18].

The annihilation time, *τ*_ann_, was determined from *Q*(*t*) as the first time at which |*Q*| < 0.1, followed by sustained loss of the skyrmion-like plateau. Subsequent finite-temperature fluctuations around *Q* ≈ 0 were not interpreted as skyrmion recovery. This distinguishes irreversible annihilation from the earlier onset of deformation.

If the annihilation criterion was not reached during a complete 63 ns record, the result was denoted *τ*_ann_ > 63 ns. This is a right-censored observation: it indicates that no annihilation occurred within that interval, not a measured thermally activated lifetime. An incomplete record is not extrapolated beyond its available endpoint.

The observed survival fraction is *P*_surv_(*t*) = *N*_surv_(*t*)/5 for five realisations observed to time *t*. The terms “stable”, “transition”, and “annihilated” are used descriptively. “Stable” denotes finite-time survival with a comparatively larger core; “transition” denotes the strongly compressed but surviving *K_u_* = 0.90 MJ/m^3^ case; and “annihilated” denotes sustained loss of the skyrmion-like charge plateau. In particular, the transition designation does not imply a lower observed survival fraction. These labels do not define equilibrium phases or long-term retention probabilities.

### 2.7. Temperature-Dependent Simulations and Velocity Analysis

Following the room-temperature screening, *K_u_* = 0.85 MJ/m^3^ was selected as a favourable compact reference condition for the temperature-dependent investigation. All five realisations survived the observation interval, while the skyrmion was less compressed than at *K_u_* = 0.90 MJ/m^3^. This selection does not establish a joint optimum in size, survival, trajectory, and dynamical precision.

The simulations were performed at *T* = 0, 100, 200, 300, 400, and 500 K, using the same geometry, magnetic parameters, current protocol, and output intervals. The temporal evolution of *Q*(*t*), *x*_sky_(*t*), and *R*_eq_(*t*) was used to characterize topology, motion, and deformation. All annihilation classifications were based on the original, unsmoothed topological-charge data.

For visualization, the topological-charge trajectories were processed using a centered moving-average filter with a window of 101 data points, corresponding to approximately 0.5 ns at the adopted 5 ps sampling interval. Before numerical differentiation, coordinate trajectories were smoothed using a third-order Savitzky–Golay filter with a window of seven data points. The velocity components and speed were then calculated as

(17) $$v_{x}\left(t\right)=\frac{dx_{\mathrm{sky}}}{\mathrm{dt}},\;v_{y}\left(t\right)=\frac{dy_{\mathrm{sky}}}{\mathrm{dt}},\;v\left(t\right)=\sqrt{v_{x}{\left(t\right)}^{2}+v_{y}{\left(t\right)}^{2}}.$$

Because 1 nm/ns = 1 m/s, the differentiated velocities were expressed directly in m/s. The confined-motion interval was 10 ≤ *t* ≤ 53 ns. The temporal statistics were defined by

(18) $$v_{\mathrm{rms}}=\sqrt{\langle v{\left(t\right)}^{2}\rangle_{t}},$$

(19) $$\sigma \left(v_{x}\right)=\sqrt{\langle {\left[v_{x}\left(t\right)-\langle v_{x}\left(t\right)\rangle_{t}\right]}^{2}\rangle_{t}},$$

(20) $$\sigma \left(v_{y}\right)=\sqrt{\langle {\left[v_{y}\left(t\right)-\langle v_{y}\left(t\right)\rangle_{t}\right]}^{2}\rangle_{t}}.$$

Here ${\left\langle \;\right\rangle }_{t}$ denotes temporal averaging over that interval, and *v*_95_ is the 95th percentile of the instantaneous speed distribution over the same samples.

The velocity statistics were evaluated separately for each realisation over 10 ≤ *t* ≤ 53 ns and subsequently averaged across the five finite-temperature simulations. The associated standard deviations describe variability between realisations; they are distinct from *σ*(*v_x_*) and *σ*(*v_y_*), which describe temporal fluctuations within a trajectory. At *T* = 0 K, the trajectory is deterministic. Temporal variations may remain under current driving, but they must not be interpreted as variability generated by different thermal seeds.

Throughout the temperature-dependent investigation, *M_s_*, *A*, and *D* were held at the values specified in Section 2.2, while *K_u_* = 0.85 MJ/m^3^. Temperature enters through the stochastic thermal field. The purpose is to isolate the response to thermal noise while retaining the same micromagnetic energy functional and current-driving protocol.

In physical ferromagnets, magnetisation, anisotropy, exchange stiffness, and DMI generally vary with temperature [43,44,45,46]. Appreciable changes can occur below the critical region [47,48], and the response also depends on film thickness and interfaces [49,50]. The present parameter set is not a temperature-dependent calibration of a specific multilayer, and no material-specific Curie temperature is assigned to it.

Accordingly, the 0–500 K sweep describes the response of a fixed-parameter model to prescribed thermal noise. It does not establish the operating-temperature range or long-term retention capability of a physical device. Predictions for a particular material require calibrated temperature-dependent properties and, near magnetic ordering, an appropriate treatment of longitudinal magnetisation dynamics [51]. The principal symbols, definitions, and units used throughout the micromagnetic model and skyrmion analysis are summarized in Table 1.

## 3. Results and Discussion

### 3.1. Room-Temperature Anisotropy Screening and Two-Stage Strategy

The first stage evaluates the anisotropy-dependent survival and compression at 300 K. The second varies the prescribed temperature at the selected *K_u_* = 0.85 MJ/m^3^ condition. The common geometry and driving protocol allow these responses to be compared within one model, while the observation window and finite number of seeds limit statistical and lifetime inferences.

### 3.2. Effect of Perpendicular Magnetic Anisotropy on Skyrmion Survival

**Figure 2:**
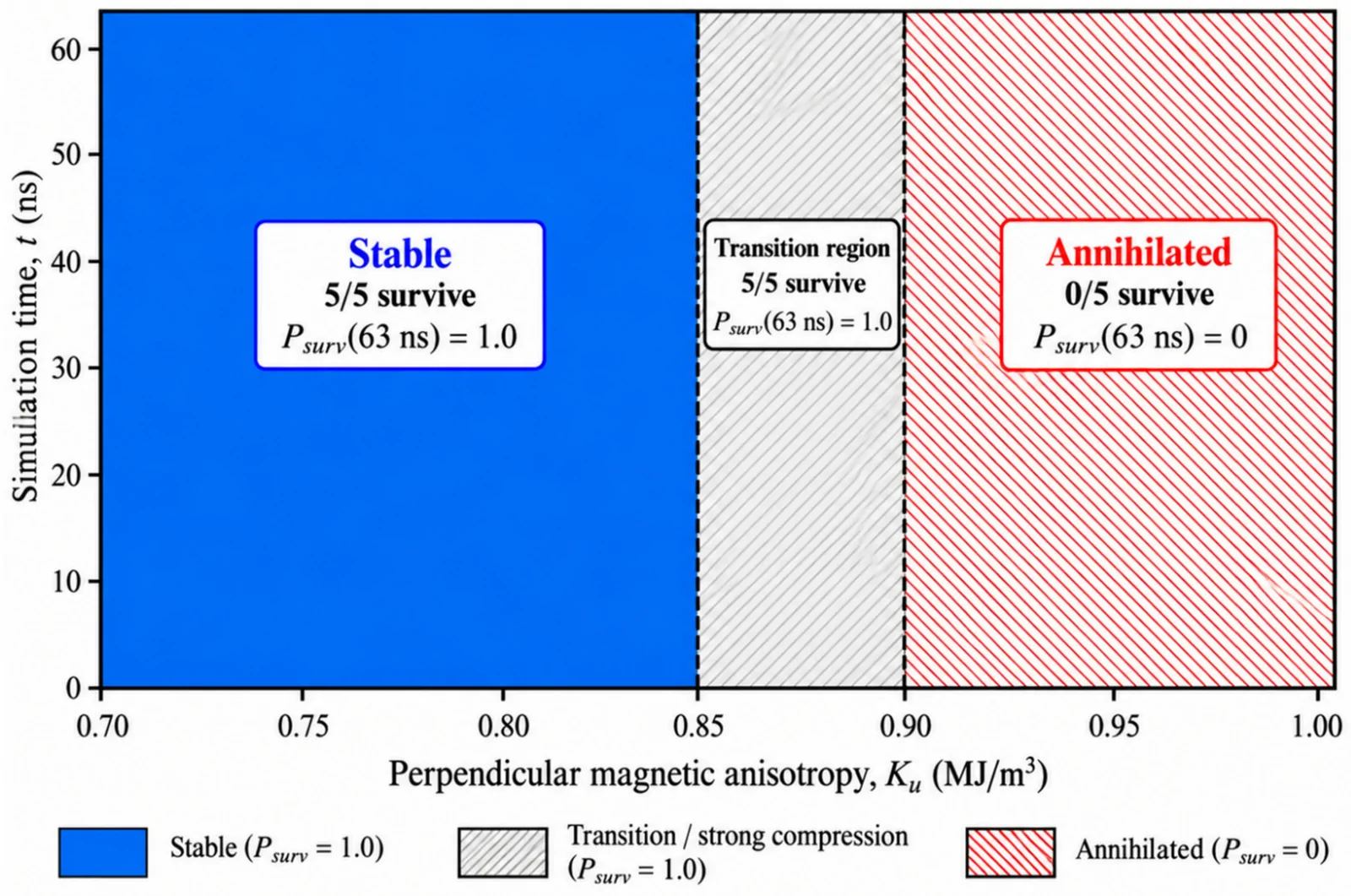
Classification of the sampled anisotropies at *T* = 300 K for *d* = 50 nm, *λ* = 5 nm, and *J* = 1.8 × 10^12^ A/m^2^. All five realisations survive to 63 ns for each sampled *K_u_* = 0.70–0.90 MJ/m^3^; all five annihilate at 0.95 and 1.00 MJ/m^3^. “Transition” denotes strong compression at 0.90 MJ/m^3^, not a smaller observed survival fraction. The coloured bands are schematic categories, not resolved continuous boundaries or the time of the annihilation event. In particular, the outcome at the sampled value 0.90 MJ/m^3^ is survival.

Figure 2 summarises the anisotropy-dependent outcomes at *T* = 300 K for *d* = 50 nm, *λ* = 5 nm, and *J* = 1.8 × 10^12^ A/m^2^. For each sampled anisotropy from 0.70 to 0.90 MJ/m^3^, all five realisations survive the 63 ns observation interval. At *K_u_* = 0.90 MJ/m^3^, the core is strongly compressed, but the observed survival fraction remains 5/5, as at the lower anisotropies.

At *K_u_* = 0.95 and 1.00 MJ/m^3^, all five realisations undergo annihilation. Thus, increasing perpendicular anisotropy does not produce a monotonic improvement in finite-time survival. The distinction between survival at 0.90 MJ/m^3^ and annihilation at the two higher values applies to the sampled conditions. Neither the exact location of a continuous boundary nor an equilibrium phase diagram is determined by this discrete sweep.

### 3.3. Effect of Perpendicular Magnetic Anisotropy on Skyrmion Survival and Annihilation Time

**Figure 3:**
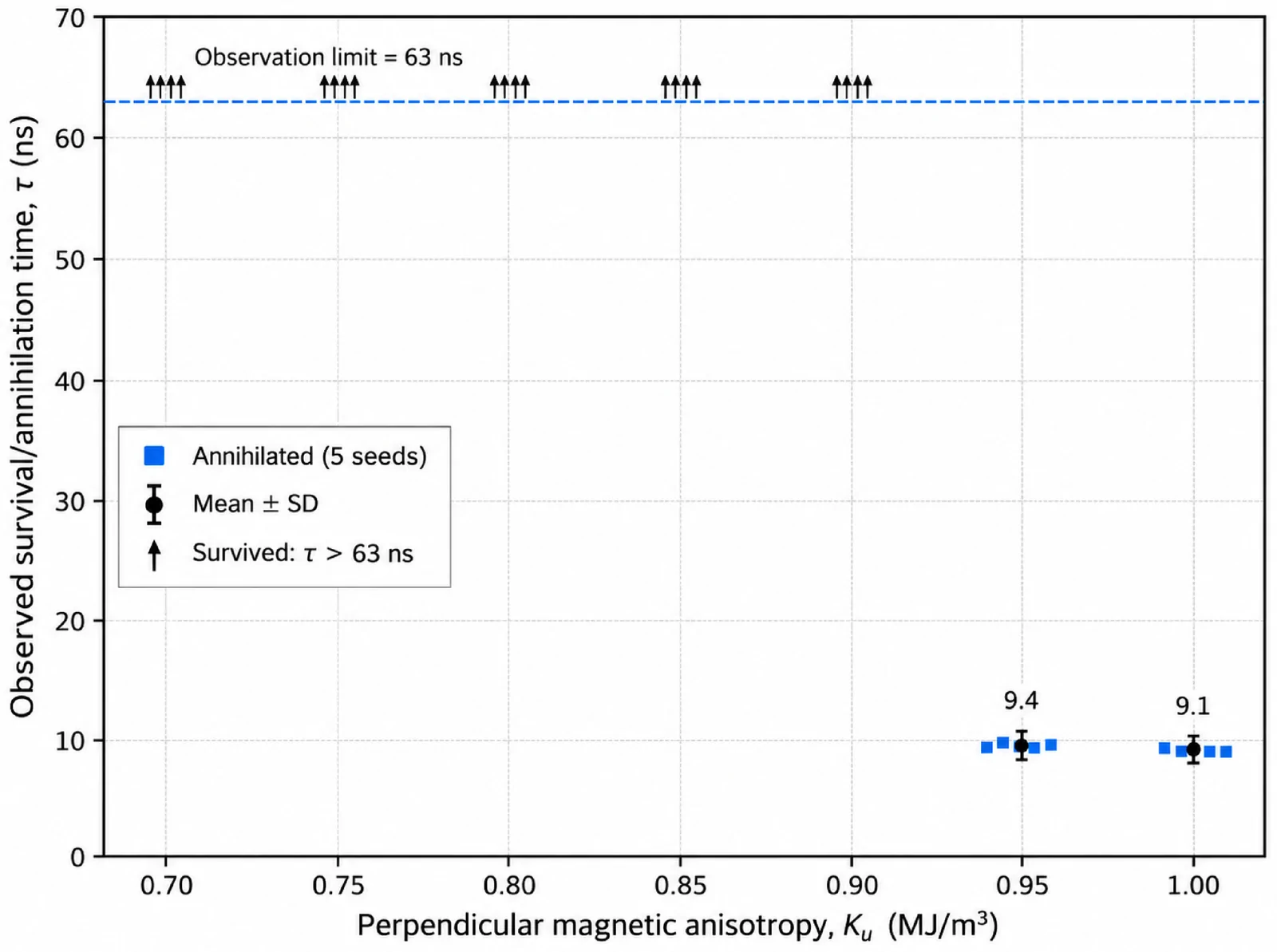
Survival observations/annihilation times at *T* = 300 K for the specified Z-junction and pulse protocol using five thermal realisations per anisotropy. Upward arrows denote no observed annihilation during 63 ns, corresponding to right-censored observations (τ > 63 ns). Blue squares denote the five annihilation times for each of the two higher-anisotropy cases. Black symbols and error bars represent the corresponding mean values and standard deviations. The horizontal dashed line indicates the 63 ns observation limit.

Figure 3 presents the individual survival and annihilation outcomes. The upward arrows for *K_u_* = 0.70, 0.75, 0.80, 0.85, and 0.90 MJ/m^3^ indicate that annihilation was not observed during the 63 ns observation interval. These cases therefore constitute right-censored observations, for which only τ > 63 ns can be established. The arrows do not assign an event time of 63 ns or establish a lifetime ranking among the surviving cases.

For *K_u_* = 0.95 and 1.00 MJ/m^3^, the five realisations show annihilation within approximately 9–10 ns. The corresponding mean values and standard deviations are calculated from the five individual annihilation times for each anisotropy. The small separation between the two high-anisotropy groups is not used to establish a statistically resolved difference in annihilation time.

These observations concern survival under the applied pulse protocol. They do not provide an activation barrier, an attempt frequency, or a thermally activated lifetime extrapolated beyond the simulated interval.

### 3.4. Evolution of the Topological Charge

**Figure 4:**
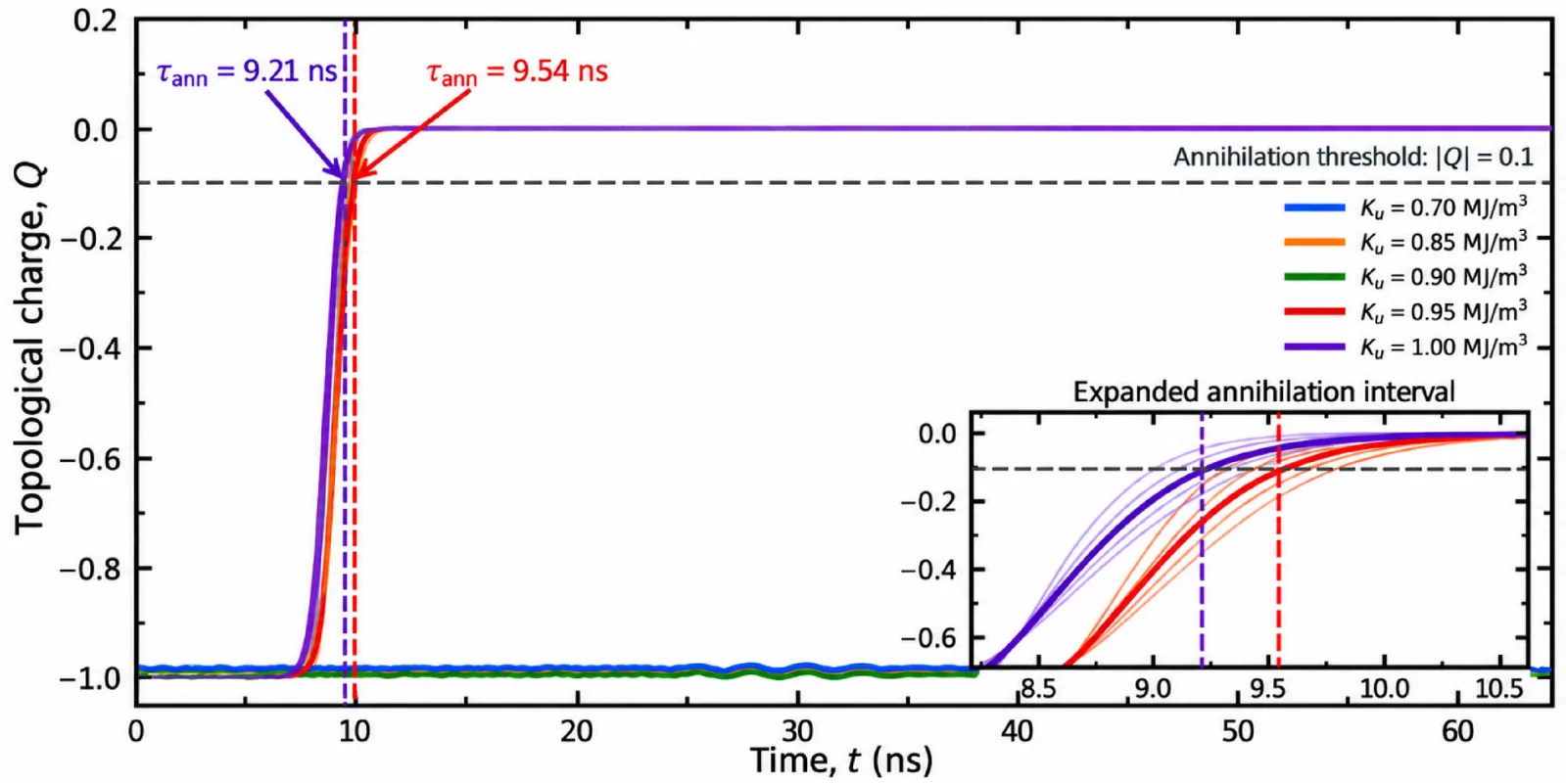
Time evolution of the topological charge for five thermal realisations at *K_u_* = 0.70, 0.85, 0.90, 0.95, and 1.00 MJ/m^3^ under the 300 K protocol. The horizontal dashed line marks *Q* = −0.1, corresponding to the adopted annihilation threshold for these initially negative-charge states. Vertical dashed lines mark reference times of 9.54 and 9.21 ns for 0.95 and 1.00 MJ/m^3^, respectively, rather than ensemble-averaged event times. The inset expands the annihilation interval.

Figure 4 shows the time evolution of the topological charge for selected anisotropies. At *K_u_* = 0.70, 0.85, and 0.90 MJ/m^3^, the trajectories remain near the skyrmion-like plateau *Q* ≈ −1. At 0.95 and 1.00 MJ/m^3^, the plateau is lost and *Q* approaches zero. The subsequent persistence near zero distinguishes annihilation from a transient deformation of an otherwise surviving skyrmion.

The vertical guides at 9.54 and 9.21 ns in the figure mark reference threshold-crossing times for 0.95 and 1.00 MJ/m^3^, respectively. They are not identified as ensemble means. The variations between the displayed trajectories do not change the qualitative distinction between survival and annihilation. The additional mesh comparison in Supplementary Figure S1 reproduces that distinction for 0.90 and 1.00 MJ/m^3^.

### 3.5. Effect of Perpendicular Magnetic Anisotropy on Skyrmion Trajectory

**Figure 5:**
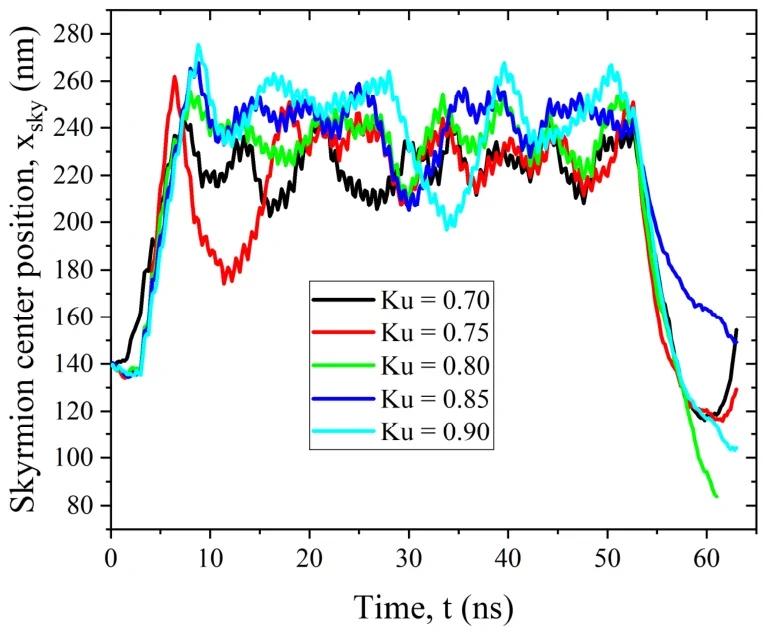
Longitudinal skyrmion-centre position for K_u_ = 0.70, 0.75, 0.80, 0.85, and 0.90 MJ/m^3^ at T = 300 K, d = 50 nm, λ = 5 nm, and J = 1.8 × 10^12^ A/m^2^. The curves represent the skyrmion-centre trajectories for the corresponding anisotropy values. Motion towards the junction is followed by confined excursions and backward relaxation after the pulse train ends near 53 ns. The K_u_ = 0.80 MJ/m^3^ position trace ends near 61 ns. No statistical uncertainty is assigned to these trajectories.

Figure 5 shows the longitudinal skyrmion-centre position for K_u_ = 0.70–0.90 MJ/m^3^. After the current is applied at approximately 3 ns, the skyrmion moves towards the Z-junction. During the interval 10 ≤ t ≤ 53 ns, it undergoes repeated forward-and-backward motion near the geometrical discontinuity rather than sustained transmission through the junction.

The amplitude and timing of these excursions vary with the anisotropy. The comparatively large excursions observed at *K_u_* = 0.90 MJ/m^3^ coexist with preservation of the topological charge. Thus, positional variation alone does not constitute evidence of annihilation. The junction-induced confinement, together with the spin-transfer torque, thermal fluctuations, and internal deformation, determines the resulting skyrmion trajectory.

After the pulse train ends near 53 ns, the skyrmion-centre position shifts towards smaller longitudinal positions, indicating backward relaxation away from the junction. The K_u_ = 0.80 MJ/m^3^ position trace ends near 61 ns and is not extrapolated to 63 ns. This shorter position record does not, by itself, determine the endpoint of the separately recorded topological-charge signal.

### 3.6. Effect of Perpendicular Magnetic Anisotropy on the Minimum Equivalent Radius

**Figure 6:**
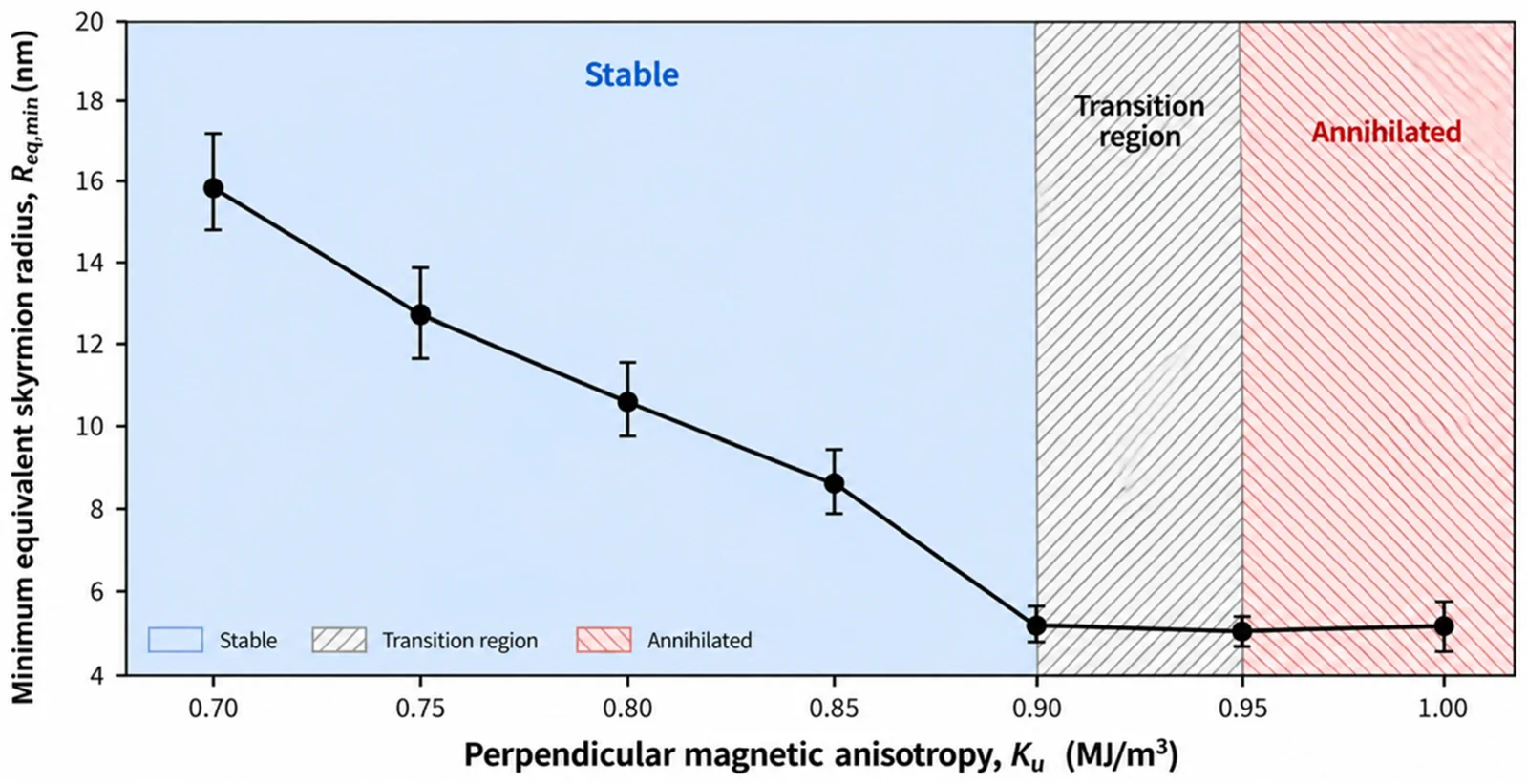
Minimum equivalent skyrmion radius versus perpendicular anisotropy at *T* = 300 K for the specified Z-junction and current protocol. Symbols and bars represent the mean minimum radii and standard deviations reported for five realisations. The strongly compressed 0.90 MJ/m^3^ case survives; the 0.95 and 1.00 MJ/m^3^ cases annihilate. For annihilating cases, the radius refers to a detectable core before loss of topology, not an equilibrium state. The shaded boundaries are schematic, and the approximately 5 nm scale is not a universal critical radius.

Figure 6 shows the reduction in minimum equivalent radius with increasing anisotropy. Between *K_u_* = 0.70 and 0.85 MJ/m^3^, the plotted radius decreases from approximately 16 to 9 nm while the skyrmion survives the observation interval. At *K_u_* = 0.90 MJ/m^3^, it reaches approximately 5.2 nm without loss of the skyrmion-like topological plateau.

The configurations at 0.95 and 1.00 MJ/m^3^ also reach small detectable radii before annihilation. Their radii are not equilibrium sizes of surviving skyrmions. The similar approximate 5 nm scale in surviving and annihilating cases shows that the radius alone is not a unique collapse criterion. Its interpretation depends on the magnetic configuration, geometry, discretisation, and definition of the detectable reversed core.

Increasing *K_u_* favours out-of-plane magnetisation and reduces the spatial extent of the reversed core. Exchange, DMI, magnetostatic interactions, and the applied field also contribute to the deformed configuration. The observed compression is therefore discussed as part of that balance, not as a measurement of a separate energy barrier or proof that DMI becomes insufficient at one universal radius. The *Q*(*t*) mesh comparison does not independently determine convergence of the numerical radius values.

### 3.7. Magnetic-Configuration Evidence of Anisotropy-Dependent Survival

**Figure 7:**
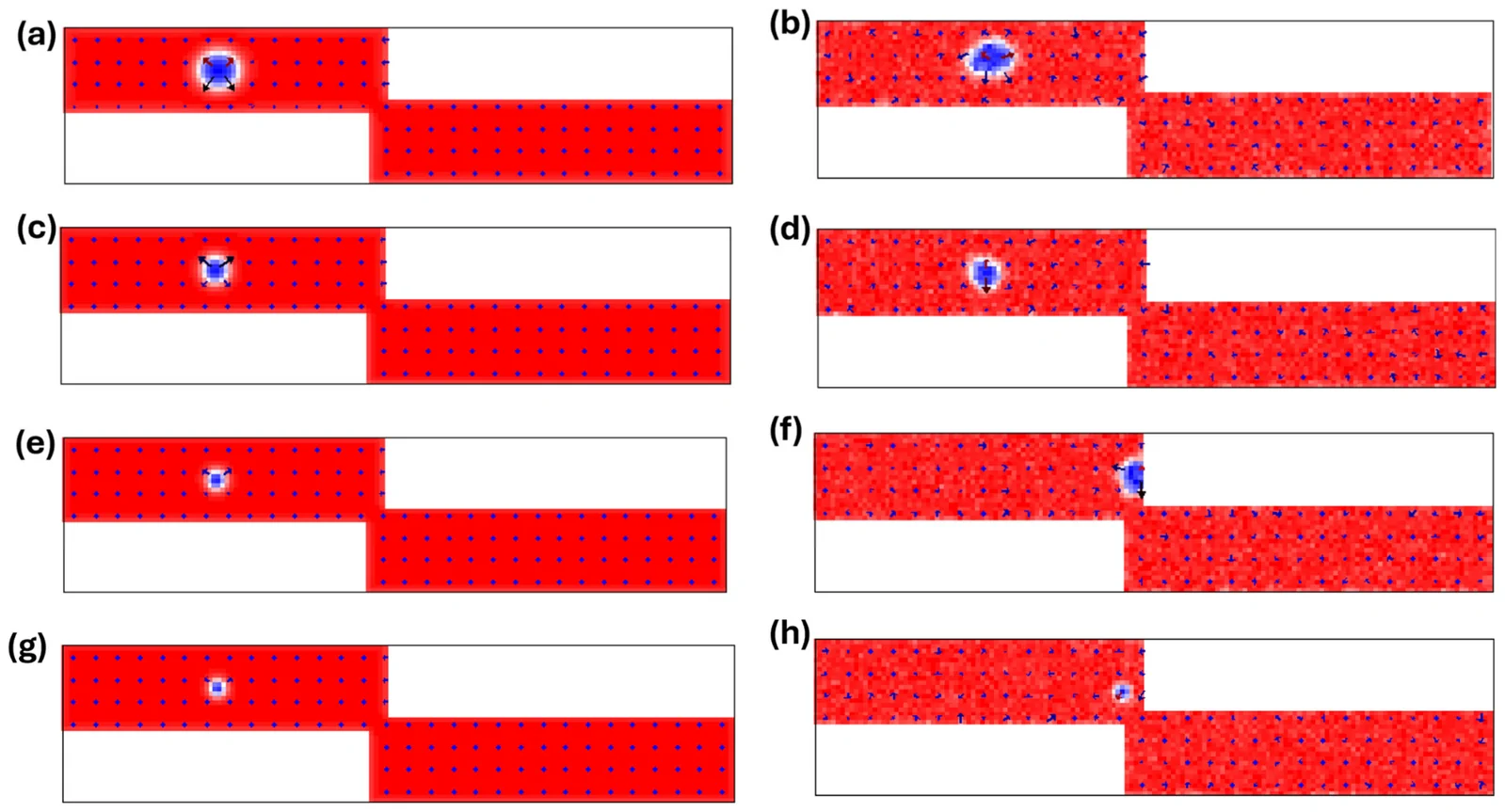
Initial and representative subsequent magnetisation configurations at *T* = 300 K, *d* = 50 nm, *λ* = 5 nm, and *J* = 1.8 × 10^12^ A/m^2^. Panels (**a**,**b**), (**c**,**d**), (**e**,**f**), and (**g**,**h**) correspond to *K_u_* = 0.70, 0.85, 0.95, and 1.00 MJ/m^3^, respectively. Panels (**f**,**h**) show deformed residual cores near the junction; they are not fully annihilated final states.

Figure 7 compares the initial and representative subsequent configurations for *K_u_* = 0.70, 0.85, 0.95, and 1.00 MJ/m^3^. The initial core becomes more compact as anisotropy increases. The subsequent configurations show deformation during interaction with the junction, including strong asymmetric compression near its inner boundary at the higher anisotropies.

Panels (f) and (h) retain localised reversed cores and therefore represent configurations near the annihilation process, not fully annihilated final states. The loss of topology is established separately by the evolution of *Q*(*t*) in Figure 4. Taken together, the configurations and charge traces are consistent with a boundary-influenced annihilation process [16,17], but do not isolate a unique microscopic route or measure its energy barrier.

The central distinction is that a skyrmion can undergo substantial compression while retaining its topology. At higher anisotropy, the current-driven interaction with the boundary can lead instead to irreversible loss of the skyrmion-like state. This interpretation does not assign a universal critical radius or an intrinsic Hall deflection as the sole origin of the junction interaction.

For surviving cases, >63 ns indicates no observed annihilation during the simulation interval. For annihilating cases, the time is defined by sustained loss of the skyrmion plateau after |*Q*| < 0.1, and the radius describes a detectable core before annihilation. “Transition” denotes strong compression with survival, not an intermediate survival fraction. Entries give the reported means and standard deviations across five realisations. The minimum equivalent skyrmion radius decreases systematically with increasing perpendicular anisotropy. The corresponding mean values and dispersions are reported consistently in Table 2 and Figure 6. For the annihilating cases, the reported radius corresponds to the minimum detectable core radius immediately before topological collapse rather than to an equilibrium radius.

### 3.8. Temperature-Dependent Skyrmion Survival and Dynamics at K_u_ = 0.85 MJ/m^3^

**Figure 8:**
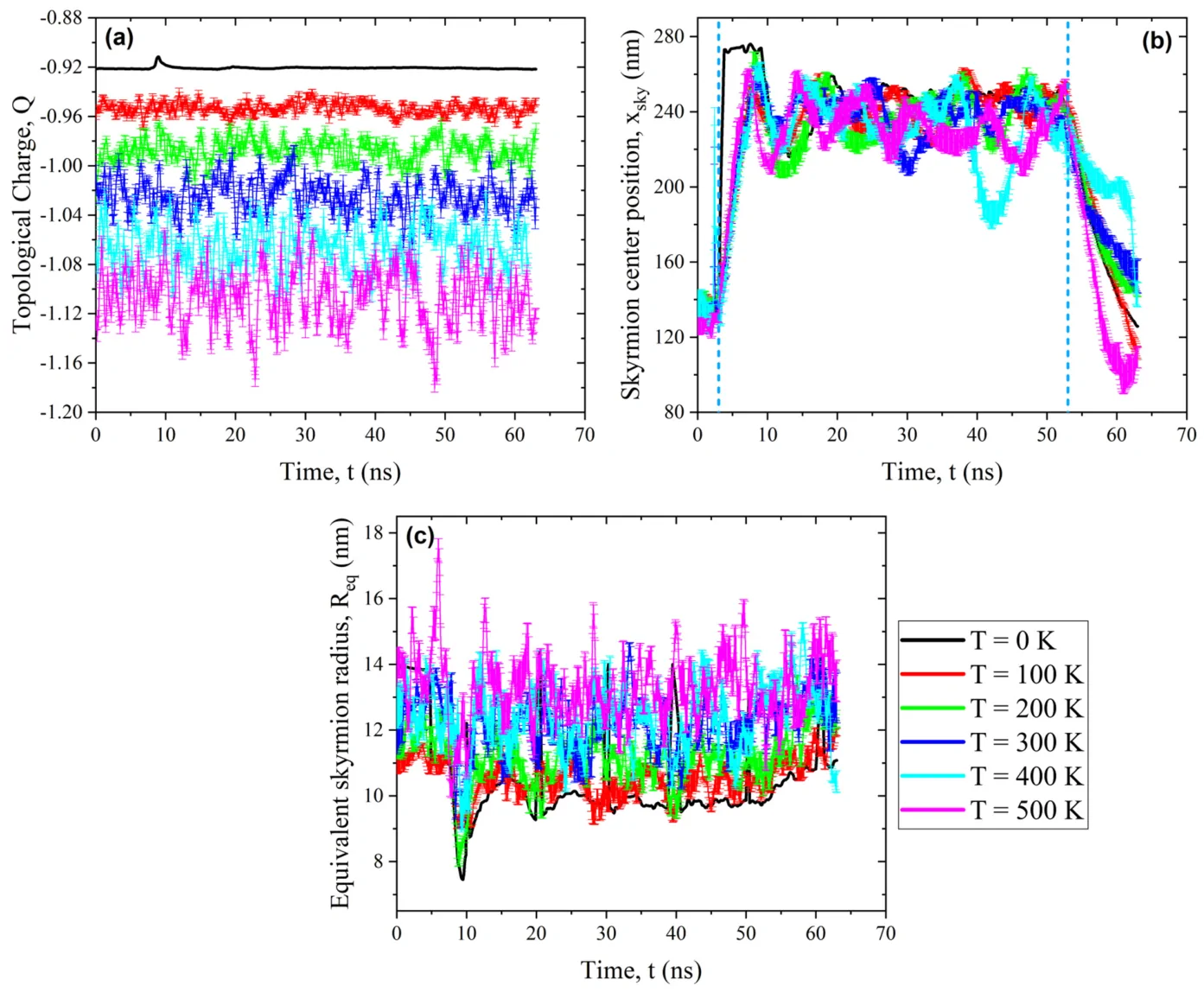
Temperature-dependent skyrmion dynamics at *K_u_* = 0.85 MJ/m^3^ in the specified Z-junction under *J* = 1.8 × 10^12^ A/m^2^. (**a**) Topological charge; (**b**) longitudinal centre position; (**c**) equivalent radius. Finite-temperature curves represent the reported means of five realisations, with bars described as their standard deviations. Vertical dashed lines in (**b**) mark 3 and 53 ns. The 400 K position trace ends near 61.9 ns.

Figure 8 presents the temperature-dependent response at the selected *K_u_* = 0.85 MJ/m^3^ condition. Panel (a) shows a skyrmion-like charge plateau throughout the available observations from 0 to 500 K, without a sustained transition to the annihilated state. The mean charge becomes more negative with increasing temperature. In a finite, discretised sample, these noninteger values should not be interpreted as additional skyrmions or as an increase in an integer topological number.

Panel (b) shows motion towards the junction followed by confined positional excursions. After the pulse train ends near 53 ns, the skyrmion relaxes backwards. The excursions become more irregular at elevated temperatures, although their detailed sequence is not monotonic in temperature. The 400 K position trace ends near 61.9 ns and is not used to infer a final position at 63 ns.

Panel (c) shows initial compression on approach to the junction followed by repeated expansion and contraction. The revised numerical summaries give a confinement-interval mean radius of approximately 10.08 nm at 0 K and 13.24 nm at 500 K. These values refer to 10 ≤ *t* ≤ 53 ns, not to the minimum radius or final relaxed configuration. The larger size variations at elevated temperature coexist with topological preservation over the observed interval.

At finite temperature, the curves and their dispersion describe the five-realisation analysis. The (T = 0) K simulation is a deterministic reference and is therefore shown without inter-realisation uncertainty. For (T > 0), the plotted curves represent the ensemble means of five independent thermal realisations, with the corresponding dispersion represented by their standard deviations. The temperature comparison is restricted to the prescribed-noise, fixed-parameter model; it is not a demonstration of experimental thermal operation up to 500 K.

### 3.9. Temperature Dependence of Skyrmion Speed and Velocity Fluctuations

**Figure 9:**
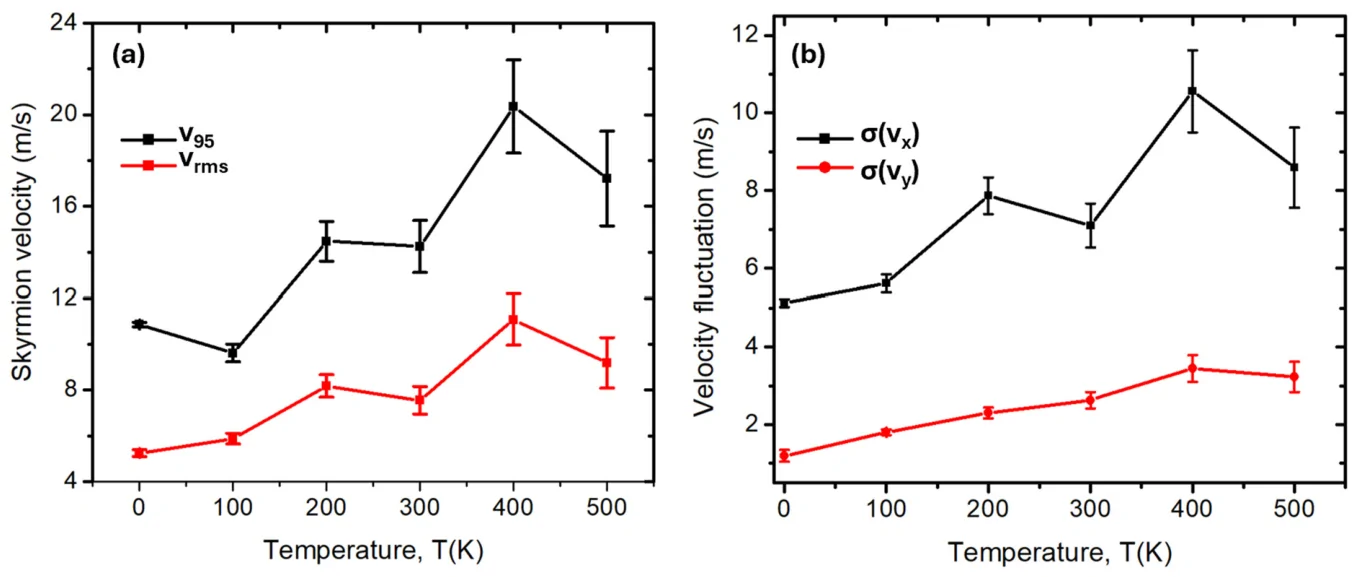
Velocity-related summaries at K_u_ = 0.85 MJ/m^3^ during the confinement interval 10 ≤ t ≤ 53 ns. (**a**) Root-mean-square speed, v_rms_, and 95th-percentile speed, v_95_. (**b**) Temporal standard deviations of the longitudinal and transverse velocity components, σ(v_x_) and σ(v_γ_), respectively. The velocity quantities are reported directly in m/s, using 1 nm/ns = 1 m/s. The T = 0 K reference corresponds to the deterministic simulation. The reported quantities characterize the temperature-dependent skyrmion dynamics over the confinement interval.

Figure 9 shows the root-mean-square speed, v_rms_, the 95th-percentile speed, v_95_, and the temporal velocity fluctuations during 10 ≤ t ≤ 53 ns. Because the confined skyrmion repeatedly changes direction, a small signed mean velocity can coexist with substantial speed and fluctuations. A larger RMS speed therefore represents stronger local motion, not necessarily more effective transport through the junction.

The displayed central values generally increase with temperature but exhibit a nonmonotonic behaviour, with their largest values at 400 K. Longitudinal fluctuations are consistently larger than transverse fluctuations throughout the temperature range. These trends characterize the temperature-dependent dynamical response of the confined skyrmion; the maximum observed at 400 K is not interpreted as evidence of a statistically established optimum or thermal resonance.

The velocity components are obtained from the temporal derivatives of the skyrmion-centre coordinates and are reported directly in m/s, using 1 nm/ns = 1 m/s, without an additional velocity normalization. The quantities σ(v_x_) and σ(v_γ_) characterize temporal fluctuations of the longitudinal and transverse velocity components, respectively.

Together, Figure 8 and Figure 9 illustrate that preservation of the skyrmion-like state can coexist with appreciable variation in position, size, and local motion. Establishing a particular coupling or resonance mechanism would require a separate time-resolved or frequency-domain analysis.

## 4. Conclusions

The influence of perpendicular magnetic anisotropy on skyrmion survival and pulsed-current-driven dynamics was investigated in a Z-junction nanotrack. For the simulated 300 K conditions, all five realisations at each *K_u_* = 0.70–0.90 MJ/m^3^ survive the 63 ns observation interval, whereas all five at 0.95 and 1.00 MJ/m^3^ annihilate within approximately 9–10 ns. Strong compression at 0.90 MJ/m^3^ coexists with topological survival. The additional mesh comparison reproduces survival at 0.90 MJ/m^3^ and annihilation at 1.00 MJ/m^3^ when the in-plane cell width is reduced from 2.0 to 0.5 nm.

The skyrmion moves towards the junction, undergoes confined positional excursions during driving, and relaxes backwards after the pulse train. The selected *K_u_* = 0.85 MJ/m^3^ condition provides a compact surviving reference for the temperature-dependent study, rather than a demonstrated joint optimum. Over the investigated temperature range, topological preservation coexists with changes in positional, radial, and velocity behaviour. The nonmonotonic velocity curves do not establish a thermal resonance.

These conclusions concern finite-time behaviour in the specified model. Survival during the simulated interval is not a measured thermally activated lifetime, and the approximate minimum-radius scale is not a universal collapse threshold. Because the magnetic coefficients are held fixed and Joule heating is not calculated self-consistently, the prescribed-temperature sweep does not determine the operating range or long-term retention capability of an experimental device.

The quantitative results apply to the investigated Z-junction with *d* = 50 nm and *λ* = 5 nm. Different junction dimensions can modify confinement, deformation, and interaction with the boundaries and may shift the anisotropy-dependent survival and annihilation outcomes. A systematic geometrical study is required before transferring the quantitative results to other designs.

The results are also specific to *J* = 1.8 × 10^12^ A/m^2^. Increasing the current strengthens the spin-transfer torque and may enhance displacement and deformation near the boundary; reducing it may weaken displacement or increase pinning. No current-density sweep was performed, so quantitative current-dependent thresholds are not inferred.

Room-temperature Néel skyrmions and current-driven motion have been demonstrated in ultrathin magnetic multilayers [10,52]. These platforms provide experimental context, but their spin–orbit-driven response is not a direct calibration of the present Zhang–Li model. In particular, a nominal 5 nm junction feature requires demanding control of edge geometry, thickness, and current distribution. Fabrication and imaging of the specific Z-junction, including the effects of edge roughness and heating, remain necessary for experimental validation.

DMI, saturation magnetisation, exchange stiffness, and damping also influence the core structure and driven response. Since these parameters were fixed, the selected anisotropy should not be regarded as universal across materials. Systematic variations in these parameters, geometry, and driving protocol are needed to establish broader design guidelines.

## Figures and Tables

**Table 1:** Principal symbols, definitions, and units used in the micromagnetic model and skyrmion analysis.

Symbol	Definition	Unit
*M_s_*	Saturation magnetisation	A/m
*A*	Exchange stiffness	J/m
*D*	Interfacial DMI constant	J/m^2^
*K_u_*	Perpendicular magnetic anisotropy constant	J/m^3^
*α*	Gilbert damping parameter	Dimensionless
*P*	Spin polarisation	Dimensionless
*ξ*	Non-adiabaticity parameter	Dimensionless
*B*_ext_	Applied magnetic flux density	T
*T*	Temperature	K
*J*	Applied current density	A/m^2^
*u*	Spin-drift velocity	m/s
*f*	Current-pulse frequency	Hz
*t*_on_	Current-on duration	ns
*t*_off_	Current-off duration	ns
*d*	Z-junction depth	nm
*λ*	Z-junction width	nm
*l*	Total nanotrack length	nm
*w*	Nanotrack width	nm
Δ*x*, Δ*y*, Δ*z*	Simulation-cell dimensions	nm
*Q*	Skyrmion topological charge	Dimensionless
*A*_sky_	Area of the reversed skyrmion core	nm^2^
*x*_sky_,*y*_sky_	Skyrmion-centre coordinates	nm
*R*_eq_	Equivalent skyrmion radius	nm
*R*_eq,min_	Minimum equivalent skyrmion radius	nm
*τ*_ann_	Skyrmion annihilation time	ns
*P*_surv_	Observed survival fraction	Dimensionless
*v_x_*, *v_y_*	Longitudinal and transverse velocity components	m/s
*v*	Instantaneous skyrmion speed	m/s
*v*_rms_	Root-mean-square skyrmion speed	m/s
*v*_95_	95th-percentile skyrmion speed	m/s
*σ*(*v_x_*)	Temporal standard deviation of longitudinal velocity	m/s
*σ*(*v_y_*)	Temporal standard deviation of transverse velocity	m/s

**Table 2:** Summary of the simulated skyrmion behaviour as a function of perpendicular magnetic anisotropy at *T* = 300 K, *d* = 50 nm, *λ* = 5 nm, and *J* = 1.8 × 10^12^ A/m^2^.

*K_u_* (MJ/m^3^)	Survival/Annihilation Time (ns)	Final *Q*	*R*_eq,min_ (nm)	Regime
0.70	>63	≈−1	15.9 ± 0.8	Stable
0.75	>63	≈−1	12.8 ± 0.7	Stable
0.80	>63	≈−1	10.7 ± 0.6	Stable
0.85	>63	≈−1	8.7 ± 0.5	Stable
0.90	>63	≈−1	5.2 ± 0.4	Transition
0.95	9.4 ± 0.22	≈0	5.0 ± 0.3	Annihilated
1.00	9.12 ± 0.19	≈0	5.0 ± 0.3	Annihilated

## Data Availability

The original contributions presented in this study are included in the article and Supplementary Material. Further inquiries can be directed to the corresponding author.

## Supplementary Materials

The following supporting information can be downloaded at https://www.mdpi.com/article/10.3390/nano16211432/s1: Figure S1: Mesh-refinement comparison of the temporal evolution of the skyrmion topological charge, Q(t), for *K_u_* = 0.90 and 1.00 MJ/m^3^ using cell sizes of 2.0 × 2.0 × 2.0 nm^3^, 1.0 × 1.0 × 2.0 nm^3^, and 0.5 × 0.5 × 2.0 nm^3^; Table S1: Individual annihilation times obtained from the five thermal realizations; Table S2: Individual minimum equivalent skyrmion radii obtained from the five thermal realizations.
