# Supplementary material for: Perpendicular Magnetic Anisotropy Engineering for Skyrmion Thermal Stability and Pulsed-Current-Driven Dynamics in a Z-Junction Nanotrack
Source: Nanomaterials (Basel). 2026 Nov 5;16(21):1432. doi: 10.3390/nano16211432 (PMC13764827; doi:10.3390/nano16211432)
Supplement: Supplementary file 1 [file nanomaterials-16-01432-s001.zip › nanomaterials-4616418-supplementary.pdf]

Supplementary Information

Perpendicular Magnetic Anisotropy Engineering for Skyrmion Thermal Stability and Pulsed-Current-Driven Dynamics in a Z-Junction Nanotrack

Mohammed Al Bahri *et al.*

In-plane mesh-refinement comparison

To assess sensitivity to the in-plane discretisation, additional simulations were performed with cell sizes of $1.0 \times 1.0 \times 2.0\ \mathrm{nm}^3$ and $0.5 \times 0.5 \times 2.0\ \mathrm{nm}^3$ and compared with the original $2.0 \times 2.0 \times 2.0\ \mathrm{nm}^3$ mesh. Figure S1 shows close agreement in the topological-charge evolution for $K_u = 0.90$ and $1.00\ \mathrm{MJ/m^3}$. All three meshes preserve the skyrmion-like plateau at $0.90\ \mathrm{MJ/m^3}$ and reproduce irreversible annihilation at approximately 9–10 ns at $1.00\ \mathrm{MJ/m^3}$. Thus, the survival and annihilation outcomes in these tested cases are robust against a fourfold reduction in the in-plane cell width. This comparison concerns $Q(t)$ and the observed topological outcome; it does not independently establish convergence of the minimum equivalent radius.

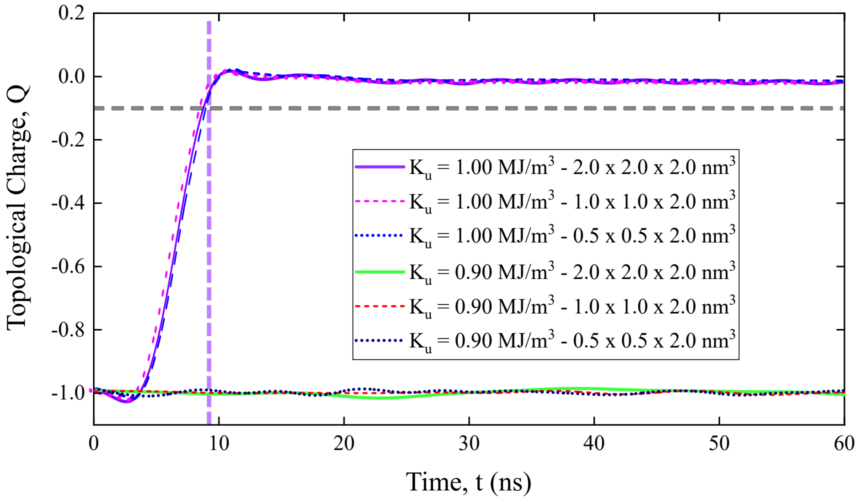

Figure S1: Mesh-refinement comparison of the temporal evolution of the skyrmion topological charge, $Q(t)$, for $K_u = 0.90$ and $1.00\ \mathrm{MJ/m^3}$ using cell sizes of $2.0 \times 2.0 \times 2.0\ \mathrm{nm}^3$, $1.0 \times 1.0 \times 2.0\ \mathrm{nm}^3$, and $0.5 \times 0.5 \times 2.0\ \mathrm{nm}^3$. The horizontal dashed line indicates $Q = -0.1$, and the vertical dashed line marks a reference annihilation time. The three meshes reproduce survival at $0.90\ \mathrm{MJ/m^3}$ and annihilation at approximately 9–10 ns at $1.00\ \mathrm{MJ/m^3}$ over the displayed 60 ns interval. The refinement concerns the in-plane cell dimensions; the cell thickness remains 2.0 nm.

Statistical analysis of the thermal realizations

To assess the statistical variability of the thermally driven skyrmion dynamics, five independent thermal realizations were considered for each value of the perpendicular magnetic anisotropy, K_u. The individual annihilation times, τ, and minimum equivalent skyrmion radii, $R_{\text{eq,min}}$, are reported in Tables S1 and S2, respectively. The mean values were calculated using the arithmetic mean, while the standard deviations were calculated using the sample standard deviation.

Table S1: Individual annihilation times obtained from the five thermal realizations.

K_u (MJ/m^3)	Seed 1 (ns)	Seed 2 (ns)	Seed 3 (ns)	Seed 4 (ns)	Seed 5 (ns)	Mean (ns)	SD (ns)
0.70	> 63	> 63	> 63	> 63	> 63	> 63	–
0.75	> 63	> 63	> 63	> 63	> 63	> 63	–
0.80	> 63	> 63	> 63	> 63	> 63	> 63	–
0.85	> 63	> 63	> 63	> 63	> 63	> 63	–
0.90	> 63	> 63	> 63	> 63	> 63	> 63	–
0.95	9.30	9.70	9.10	9.50	9.40	9.40	0.22
1.00	9.10	9.40	8.90	9.20	9.00	9.12	0.19

For $K_u = 0.70$–0.90 MJ/m^3, > 63 ns indicates that no annihilation was observed within the 63 ns simulation window. The mean and standard deviation for the annihilating cases were calculated from the five individual realizations using the arithmetic mean and sample standard deviation.

Table S2: Individual minimum equivalent skyrmion radii obtained from the five thermal realizations.

K_u (MJ/m^3)	Seed 1 (nm)	Seed 2 (nm)	Seed 3 (nm)	Seed 4 (nm)	Seed 5 (nm)	Mean (nm)	SD (nm)
0.70	14.90	15.40	15.90	16.40	16.90	15.90	0.83
0.75	11.90	12.35	12.80	13.25	13.70	12.80	0.71
0.80	9.90	10.30	10.70	11.10	11.50	10.70	0.63
0.85	8.00	8.35	8.70	9.05	9.40	8.70	0.54
0.90	4.70	5.00	5.20	5.40	5.70	5.20	0.38
0.95	4.60	4.80	5.00	5.20	5.40	5.00	0.32
1.00	4.60	4.80	5.00	5.20	5.40	5.00	0.32

The minimum equivalent skyrmion radius, $R_{\text{eq,min}}$, was determined from the minimum radius reached during the simulated evolution. The mean values were calculated as the arithmetic mean of the five realizations, and the standard deviations were calculated using the sample standard deviation.
